# Cross Interaction between M2 Muscarinic Receptor and Notch1/EGFR Pathway in Human Glioblastoma Cancer Stem Cells: Effects on Cell Cycle Progression and Survival

**DOI:** 10.3390/cells9030657

**Published:** 2020-03-09

**Authors:** Ilaria Cristofaro, Francesco Alessandrini, Zaira Spinello, Claudia Guerriero, Mario Fiore, Elisa Caffarelli, Pietro Laneve, Luciana Dini, Luciano Conti, Ada Maria Tata

**Affiliations:** 1 Department of Biology and Biotechnologies Charles Darwin, Sapienza, University of Rome, 00185 Rome, Italy; ilaria.cristofaro@uniroma1.it (I.C.); francesco.alessandrini@northwestern.edu (F.A.); zaira.spinello@uniroma1.it (Z.S.); Claudia.Guerriero@uniroma1.it (C.G.); luciana.dini@uniroma1.it (L.D.); 2 IBPM, Institute of Molecular Biology and Pathology, CNR, 00185 Rome, Italy; mario.fiore@uniroma1.it (M.F.); Elisa.caffarelli@gmail.com (E.C.); Pietro.laneve@uniroma1.it (P.L.); 3 Department of Cellular, Computational and Integrative Biology-CIBIO, University of Trento, 38123 Trento, Italy; Luciano.Conti@unitn.it; 4 Research center of Neurobiology, Sapienza, University of Rome, 00185 Rome, Italy

**Keywords:** cancer stem cells, M2 muscarinic receptors, EGFR, Notch, cell cycle, apoptosis

## Abstract

Glioblastomas (GBM) are the most aggressive form of primary brain tumors in humans. A key feature of malignant gliomas is their cellular heterogeneity. In particular, the presence of an undifferentiated cell population of defined Glioblastoma Stem cells (GSCs) was reported. Increased expression of anti-apoptotic and chemo-resistance genes in GCSs subpopulation favors their high resistance to a broad spectrum of drugs. Our previous studies showed the ability of M2 muscarinic receptors to negatively modulate the cell growth in GBM cell lines and in the GSCs. The aim of this study was to better characterize the inhibitory effects of M2 receptors on cell proliferation and survival in GSCs and investigate the molecular mechanisms underlying the M2-mediated cell proliferation arrest and decreased survival. Moreover, we also evaluated the ability of M2 receptors to interfere with Notch1 and EGFR pathways, whose activation promotes GSCs proliferation. Our data demonstrate that M2 receptors activation impairs cell cycle progression and survival in the primary GSC lines analyzed (GB7 and GB8). Moreover, we also demonstrated the ability of M2 receptor to inhibit Notch1 and EGFR expression, highlighting a molecular interaction between M2 receptor and the Notch-1/EGFR pathways also in GSCs.

## 1. Introduction

Malignant gliomas are the most common type of primary malignant brain tumor, with an annual incidence of 5.6 per 100,000 individuals. This pathology is most common in the sixth through eighth decades of life. The World Health organization (WHO) groups gliomas into four histological grades based on degrees of differentiation, anaplasia and aggressiveness: (I) grade I or pilocytic astrocytoma, (II) grade II or low-grade astrocytoma, (III) grade III or anaplastic astrocytoma, and (IV) grade IV or glioblastoma multiforme (GBM) [1].

Among these, GBM is the most frequent and malignant histological type, accounting for 65% of gliomas. The median survival of patients diagnosed with GBM is 12–15 months and the 5 years survival rate for GBM patients is less than 5%.

The standard therapeutic regimen for GBM includes chirurgic resection, followed by concurrent radio- and chemo-therapy, including the treatment with the alkylating agent Temozolomide (TMZ) [2,3]. Unfortunately, these therapeutic treatments do not allow to eradicate completely the tumor and, as a result, are inefficient especially in counteracting the undifferentiated tumor cells growth. In fact, in GBM, such as in other solid tumors, the presence of an undifferentiated tumor cell subpopulation named “Cancer Stem Cells” (CSCs) was shown [4,5]. The properties of CSCs include self-renewal; the capability to induce oncogenesis in immunosuppressed xenografts recipients; the ability to enhance proliferation, invasiveness, migration, metastasis, and resistance to drugs and radiation; and finally the competence to differentiate, giving rise to heterogeneous cell populations establishing the tumor bulk [4,5,6].

In the Glioblastomas Cancer Stem Cells (GSCs), Notch and Epidermal Growth Factor receptor (EGFR) pathways are among the most frequent components involved in cell proliferation and survival [7]. The EGF pathway is a landmark of classical GBM subtype and one of the most important signaling pathways that regulate growth, proliferation, migration, and cell survival through various inter-acting downstream effectors [8,9].

The Notch pathway is involved in important cellular function such as proliferation, differentiation and maintenance of stem cell properties [10].

Muscarinic receptors belong to the class of the G-protein coupled receptors (GPCRs) [11]. These are expressed in several tumors (e.g., colon, ovary, lung, breast cancer) and are involved in tumor cell proliferation and migration [12,13,14]. Muscarinic receptors are expressed in astrocytes and low grade astrocytomas, and their activation modulates cell proliferation and migration [15]. In our previous studies, we demonstrated that GBM cells express different muscarinic receptor subtypes, and that the stimulation of M2 receptor subtype by selective M2 agonist *arecaidine propargyl ester* (APE), decreases cell proliferation and survival in GBM cell lines and in primary cell cultures [16,17]. Recently, we also demonstrated that the selective activation of M2 receptors by APE or dualsteric agonist N8-Iperoxo inhibits cell growth in GSCs obtained from two different human tumor biopsies (GB7 and GB8 cells) [18,19]. In order to better understand the mechanisms underlying the decreased cell proliferation and survival, in the present work we described the ability of APE to differently modulate the cell cycle progression in GB7 and GB8 cells. Moreover, the cross-interaction between M2 receptors and Notch1/EGFR pathways has also been investigated, demonstrating that the APE-induced decreased cell proliferation is dependent on the impaired activity of these two signaling pathways.

## 2. Materials and Methods

### 2.1. Cell Cultures

The glioblastoma cancer stem cell lines (GSCs) GB7 and GB8 were obtained from human biopsies [5,20]. The cells were cultured on a laminin-coated plastic (1 μg/mL, Sigma-Aldrich, St. Louis, MO, USA) or as neurospheres (in uncoated plastic) and maintained in Euromed-N medium (EuroClone, Milan, Italy) supplemented with 1% streptomycin, 50 IU/mL penicillin, (Sigma-Aldrich, St. Louis, MO, USA), 1% glutamine (Sigma-Aldrich, St. Louis, MO, USA), 1% N2 supplement (Invitrogen, Monza, Italy), 2% B27 (Invitrogen, Monza, Italy), 20 ng/mL EGF (Recombinant Human Epidermal growth factor, Peprotec, London, UK), and 20 ng/mL FGF (Recombinant Human FGF-basic, Preprotech, London, UK). The cell cultures were maintained at 37 °C in an atmosphere of 5% CO_2_/95% air.

### 2.2. Pharmacological Treatments

M2 agonist arecaidine propargyl ester hydrobromide (APE) was used to selectively stimulate the M2 muscarinic receptor subtype. The ability of this agonist to bind the M2 receptor subtype was previously demonstrated in GBM established cell lines (U87 and U251) and in GSCs (GB7 and GB8 cells) by pharmacological binding experiments and knockdown of the receptors by siRNA transfection pool [17,18].

Epidermal Growth Factor receptor (EGFR) tyrosine kinase inhibitor (TKI) N-(3-chlorophenyl)-6,7-dimethoxy-4-quinazolinamine), tyrphostin AG1478 (Sigma-Aldrich, St. Louis, MO, USA) was used at final concentration of 1 µM, to inhibit the EGFR pathway [21].

### 2.3. Immunocytochemistry

GB7 cells were plated onto 35-mm-diameter dishes in complete medium. Then, the cells were rinsed with phosphate buffer saline (PBS) pH 7.4, fixed with 4% paraformaldehyde for 20 min at room temperature (RT), washed in PBS, and permeabilized by treatment with blocking buffer (0.1% Triton X-100, 10% NGS in PBS) for 1 h at RT. The cells were then incubated overnight at +4 °C with anti-Nestin (1:200, Abcam, Cambridge, UK), anti-CD133 (1:100, Miltenyi Biotec, Teterow, Germany), anti-REST (1:200, Abcam, Cambridge, UK) antibodies diluted in antibody incubation buffer (0.1% Triton X-100, 1% NGS, 1% BSA in PBS). The next day, after three washes with PBS, the cells were incubated for 1 h at RT with a goat anti-mouse-Alexa 594-conjugated (1:2000, Promega, Madison, WI, USA) or goat anti-rabbit-Alexa 488-conjugated (1:2000 Promega, Madison, WI, USA) secondary antibodies diluted in incubation buffer. After washing in PBS, the cells were finally mounted with 30 μL of Anti Fade Mounting Medium with DAPI (Immunological Science, Rome, Italy). Negative controls were obtained by omitting the primary antibodies (data not shown).

### 2.4. RNA Extraction and RT-PCR Analysis

Total RNA was extracted by using Cultured Cell Total RNA Extraction Mini Kit (FMB, PA, USA) following the manufacturer’s instructions. RNA samples (2 μg) were reverse transcribed for 60 min at 37 °C with Random Primers (Promega, Madison, WI, USA) and M-MLV reverse transcriptase (Promega, Madison, WI, USA). Then, PCR reagents, primers, and GoTaq Green Master Mix (Promega, Madison, WI, USA) were added to each reaction tube. The expression of the transcripts was evaluated by semi-quantitative RT-PCR analysis using the following primers:

**M2**: forward, 5′-CCAAGACCCCGTTTCTCCAAG-3′;

  reverse, 5′- CCTTCTCCTCTCCCCTGAACAC-3′.

**Nestin:** forward, 5′-TGCGGGCTACTGAAAAGTTC-3′

  reverse, 5′-TGTAGGCCCTGTTTCTCCTG-3′

**Sox2**: forward, 5′-ACACCAATCCCATCCACACT-3′

  reverse, 5′-GCAAACTTCTTGCAAAGCTC-3′

**CD133**: forward 5′-GCATTGGCATCTTCTATGGTT-3′

  reverse, 5′-CGCCTTGTCCTTGGTAGTGT-3′

**REST:** forward 5′-ACTTTGTCCTTACTCAAGTT-3′

  reverse 5′-GCATGGCGGGTTACTTCAT-3′

**Hes1:** forward, 5′- ATGACAGTGAAGCACCTCCG- 3′;

  reverse, 5′ - AGGTCATGGCATTGATCTGG- 3′

**Notch1**: forward, 5′ AGGCATCATGCATGTCAAAC - 3′;

  reverse, 5′ - TGTGTTGCTGGAGCATCTTC - 3′

**Notch2**: forward, 5′ TTGTGTGAACAATGGGCAGT - 3′;

  reverse, 5′ - TTCATAGCCATTCGGGTGAT - 3′

**Notch3**: forward, 5′- CATCTGGTTGCTGCTGACAT-3′

  reverse, 5′- ATCAGGTCGGAGATGATGCT-3′

**Egfr:** forward, 5′- AGCATGTCAAGATCACAGAT - 3′;

  reverse, 5′ - TGGATCCAAAGGTCATCAA - 3′

**ErbB3:** forward, 5′- GGAGTCTTGCCAGGAGTCT-3′

  reverse 5′- AGGAGTCAGCAGACTGTGG-3′

**18S:** forward, 5′-CCAGTAAGTGCGGGTCATAAGC -3′;

  reverse, 5′-AACGATCCAATCGGTAGTAGCG -3′

PCR conditions were: initial denaturation at 95 °C for 3 min; 35 cycles with the following profile: 95 °C for 30 s, 60 °C for 30 s and 72 °C for 30 s; and final extension at 72 °C for 5 min. For each PCR reaction, 25 μL samples were run on 3% agarose gel. 18 S transcript level was used as a housekeeping gene.

For miR-34-5p expression, 6 ng of cDNA, synthesized through the miScript II Reverse Transcription kit (cat. n. 218161, Qiagen, Milan, Italy), were used as a template for the qRT-PCR reaction. Amplifications were performed through the miScript-SYBR green PCR kit (cat. n. 218073, Qiagen) on a 7500 Fast Real-Time PCR (Applied Biosystems Italia, Monza, Italy). Commercially available DNA oligonucleotides were used as primers for the detection of miR-34-5p (Hs_miR-34a_1, cat. n. MS00003318, Qiagen) and as a control of snRNA U6 (Hs_RNU6B_13, cat. n. MS00014000, Qiagen). Relative quantification was performed using the comparative ΔΔCT method.

### 2.5. Western Blot Analysis

To detect the expression of M2 receptor, Notch1 and EGFR, protein extracts were run on 10% SDS-polyacrilamide gel (PAGE) and transferred to PVDF membranes (Merck Millipore, Darmstadt, Germany) and blocked for 1 h in 5% non-fat milk powder (Sigma-Aldrich, St. Louis, MO, USA) in PBS containing 0.1% Tween-20, and then incubated with monoclonal anti-M2 (1:800 Abcam, Cambridge, UK), anti-Notch (1:200, Santa Cruz Biotechnologies, Dallas, TX, USA), anti-PCNA (1:700, Sigma-Aldrich, St. Louis, MO, USA), anti-p53 (1:100; Santa Cruz, Temecula, CA, USA) and anti-EGFR (1:1000, Merck Millipore, Darmstadt, Germany) primary antibodies, overnight at 4 °C. Then the blots were washed and incubated for 1 h at RT with secondary antibodies horseradish-peroxidase-conjugated (1:20000 Promega, Madison, WI, USA). The reaction was revealed by ECL chemiluminescence reagent (Euroclone, Milan, Italy). The immunoreactive signal was revealed by exposure to Chemidoc (Molecular Imager ChemiDoc XRS+ System with Image Lab Software, Biorad, CA, USA), and band intensities were quantified by optical density using ImageJ software (National Institutes of Health). GAPDH and β-actin were used as reference proteins (loading control).

### 2.6. Flow Cytometry Analysis

The cells were plated onto T25 flask at a density of 4 × 10^4^ cells/cm^2^. The day after plating, the cells, excluding control samples, were treated with 10^−4^ M APE agonist for 72, 96 and 120 h. At the end of the treatment, cells were incubated for 90 min with 45 μM bromodeoxyuridine (BrdUrd, Sigma-Aldrich, St. Louis, MO, USA), collected by trypsinization, centrifuged for 3 min at 1500 RPM, washed with PBS for tree times, and then fixed in methanol/PBS (1:1; *v*/*v*).

To identify cells in S phase, DNA content and BrdU incorporation were determined by staining with propidium iodide (PI) and anti-BrdU antibody, respectively. DNA was denaturated by incubating the cells in 3N HCl for 45 min at RT, followed by neutralization with 0.1 M sodium tetraborate. Samples were then incubated with monoclonal anti-BrdU antibody (1:50 *v*/*v*; Dako, MI, Italy) for 1 h at RT, washed twice with 0.5% Tween-20 in PBS and incubated for 45 min with goat anti-mouse Alexa fluor 488-conjugated antibody (1:1200; Invitrogen Monza, Italy). Samples were washed twice with PBS and stained with propidium iodide (10 μg/mL) for 15 min at RT. Flow cytometry analysis was performed with a flow cytometer Coulter Epics XL with 488 nm wavelength excitation, and 10^4^ events were collected for each sample. To evaluate the apoptotic cells, Annexin V kit was used following the manufacturer’s instructions (Immunological Sciences, Rome, Italy).

To identify the cells with DNA damage, the samples were incubated with monoclonal antibody directed against phospho-γH2AX (Millipore, MI, Italy) for 1 h at RT, washed, and incubated for 45 min with anti-mouse Alexa fluor 488-conjugated antibody (1:1200; Invitrogen Monza, Italy). Monoparametric (DNA histograms) and biparametric (BrdU content vs. DNA content) analyses were obtained using WinMDI 2.9 software (Scripps Research Institute, La Jolla, CA, USA).

### 2.7. Cell Death Analysis

Cell death was evaluated by flow cytometry analysis using propidium iodide (PI) staining. Cells were plated into flask T25 at density of 4 × 10^4^ cells/cm^2^ and the day after treated with 10^−4^ M APE for 72, 96, and 120 h for GB8 cells and 48, 72, and 96 h for GB7 cells. Then, the cells were collected, suspended in 2 mL of PBS buffer containing 0.1% Triton X-100 (Sigma-Aldrich, St. Louis, MO, USA), incubated for 5 min at RT, and subsequently stained with PI (10 μg/mL) and analysed by using a Coulter Epics XL flow cytometer. For each sample, 10^4^ events were recorded. Cells with a hypodiploid DNA content and a higher granularity (SSC) at G0-G1 phase (sub-G1) were quantified as dead cells [22,23].

### 2.8. Statistical Analysis

Student’s t test and one-way ANOVA test followed by Bonferroni’s post test were used to evaluate statistical significance within the different samples. The results were considered statistically significant at *p* < 0.05 (*), *p* < 0.01 (**), *p* < 0.001 (***), and *p* < 0.0001 (****).

## 3. Results

### 3.1. Different GSC Lines Exhibit Different M2 Muscarinic Receptor Expression Levels

**Figure 1:**
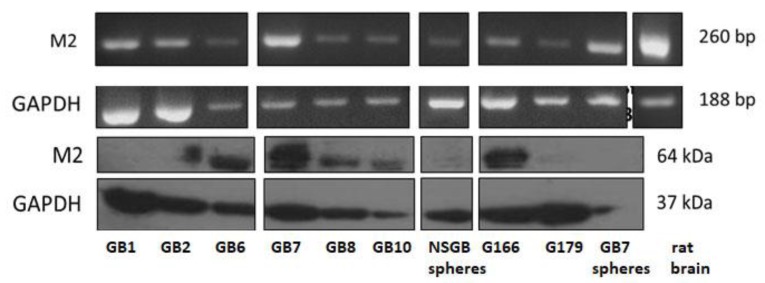
M2 protein expression in different GSC lines. Upper panels RT-PCR analysis; Lower panels Western blot analysis. GAPDH was used as an internal reference for transcript and protein analysis.

The presence of M2 muscarinic receptors was investigated in several GSC lines derived from different patients’ biopsies. Semi-quantitative RT-PCR analysis showed that all GSCs express the M2 transcript, albeit different levels of expression were evident (Figure 1 upper panels). Western blotting analysis was also performed in the same GSC lines, indicating that only some of these express M2 receptor at the protein level (i.e., GB6, GB7, GB8, GB10, and GB166) (Figure 1 lower panels). For subsequent studies, we selected GB7 and GB8 cells, considering their different levels of M2 receptor expression. In fact, the levels of M2 receptor expression appeared significantly higher in GB7 than in GB8 cells (GB7>GB8) [18]. In particular, the expression of M2 receptor protein in GB7 cells was also evaluated in GB7 grown in adherent or neurosphere conditions, confirming that the expression of M2 receptor was present in both culture conditions (see Supplementary Figure S1).

### 3.2. GB7 and GB8 Cells Express Stemness Markers and Components of Notch/EGFR Molecular Pathways

**Figure 2:**
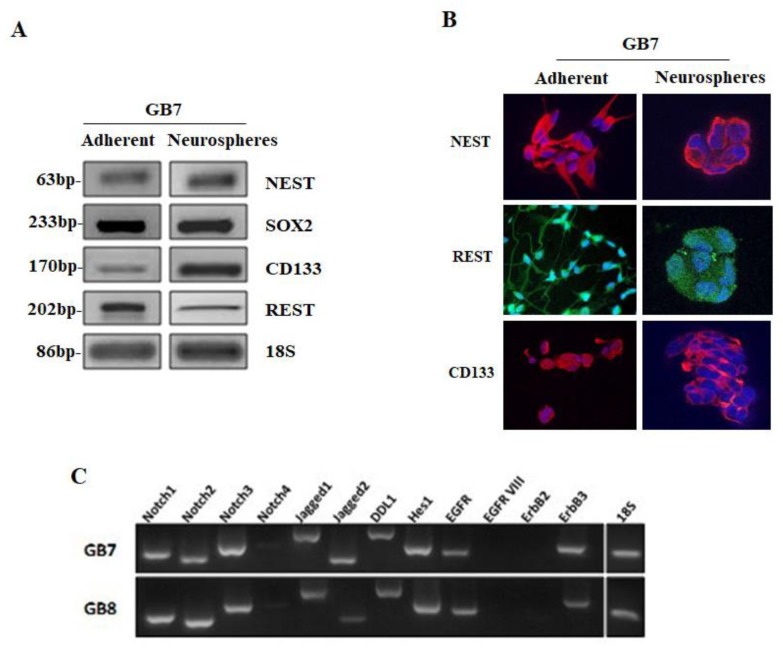
GB7 cells grown in adherent and as neurospheres. (**A**) RT-PCR analysis of stemness markers Nestin (NEST), SOX2, CD133, and REST. 18 S was used as a housekeeping gene. (**B**) Immunostaining for NESTIN, REST and CD133 in GB7 cells grown as a monolayer and as neuropheres in floating conditions (magnification × 100). (**C**) RT-PCR analysis for gene expression for different genes involved in Notch and EGFR pathways in GB7 and GB8 cells. 18 S was used as a housekeeping gene.

The expression of stemness markers (i.e., REST, CD133, Nestin and Sox2) in the two cell lines selected for our subsequent experiments was evaluated by semi-quantitative RT-PCR analysis. GSCs were cultured in adherent and as neurospheres in floating conditions (Figure 2A). In Figure 2, only the results obtained for GB7 cells are reported, nevertheless, similar results have been obtained also for the GB8 cell line (see Supplementary Figure S2 and [18]). The presence of REST, CD133 and NESTIN was also investigated at protein level by immunocytochemistry (Figure 2B). The results confirmed that the stem cell markers were expressed in GB7 cells grown both as an adherent and as neurosphere cultures. We also investigated the expression of Notch/ Delta and EGFR family members by RT-PCR analysis. We found that the transcripts for different members of Notch pathway (Notch1, Notch2, Notch3, Delta like1, Jagged 1, Jagged 2, and Hes1) were expressed both in GB7 and GB8 cells (Figure 2C). Similarly, also EGFR and ErbB3 receptors appeared to be expressed in both GSC lines. Interestingly, the mutated form of EGFR (Variant III), usually expressed in different GBM and breast cancers [24], did not appear to be expressed in either of the two GSC lines considered (Figure 2C).

### 3.3. Activation of M2 Muscarinic Receptor Differently Affects Cell Growth and Survival in GB7 and GB8 Cells

Previous data showed that APE treatment was able to significantly inhibit cell growth in GB7 cells in a time- and dose-dependent manner, while in GB8 cells, only higher doses of APE (100 μM) were able to affect cell growth, in a time-dependent manner [18].

**Figure 3:**
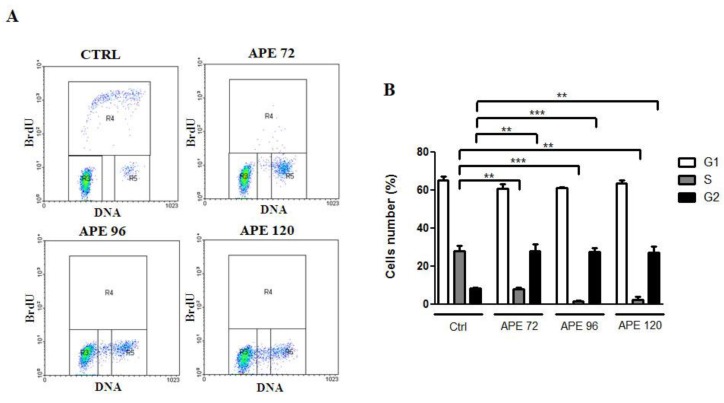
(**A**) Bivariate analysis of BrdUrd incorporation and DNA content in GB7 cells at 72, 96 and 120 h after 100 μM APE treatment. (**B**) Percentage of GB7 cells in G1, S, and G2/M phases after 100 μM APE treatment. (t-test; **p < 0.01; ***p < 0.001). The data are the average ±SEM of three independent experiments.

To evaluate whether the APE-induced decrease in cell number was dependent on the impaired cell proliferation or survival, we performed a cell cycle analysis on GB7 and GB8 cells treated with 100 μM APE for 72, 96 and 120 h. Before collecting the cells, BrdU incorporation was performed for 90 min to monitor the S phase progression. The flow cytometry analysis has been performed selecting specific gates that have allowed to analyze the distribution of the cells in different cell cycle phases (G1-S-G2/M). The bi-parametric analysis of BrdU labeling versus DNA content allowed to evaluate both cell progression through the G1/S/G2-M phases and the identification of cells in S phase. This analysis showed that GB7 cells exhibited a significant decrease in the BrdU labelled cell fraction after APE treatment accompanied by a decrease in the percentage of cells in S phase and accumulation of cells in the G2/M phase (Figure 3A,B).

**Figure 4:**
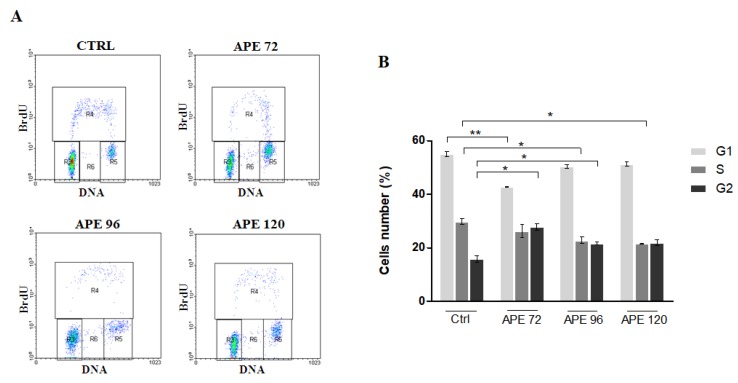
(**A**) Bivariate analysis of BrdU incorporation and DNA content in GB8 cells at 72, 96 and 120 h after 100 μM APE treatment. (**B**) Percentage of GB8 cells in G1, S, G2/M phases after 100 μM APE treatment. (t-test; *p < 0.05; **p < 0.01). The data are the average ±SEM of three independent experiments.

**Figure 5:**
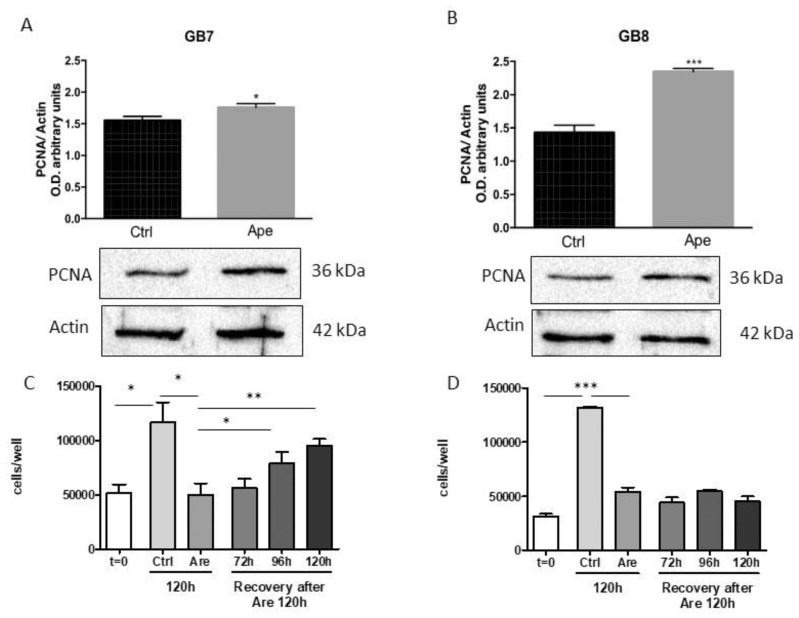
Western blot analysis for PCNA expression in GB7 (**A**) and GB8 (**B**) cells in absence (ctrl) and presence of 100 µM APE (48 h). The diagrams above report the densitometric analysis of the PCNA immunoreactive bands normalized to those of the β-actin reference. The data are the average ±SEM of three independent experiments (t-test; *p < 0.05; *** *p*< 0.01). (**C**) and (**D**) Recovery of cell proliferation after APE withdrawal. The cells were maintained for 120 h in the presence of APE 100 µM. Then the APE was removed from the culture media and substituted by fresh medium without APE for an additional 72, 96 and 120 h. Results are reported for GB7 (**C**) and GB8 (**D**) cells. (t-test; *p < 0.05; ** *p* < 0.01; ***p < 0.001).

Instead, FACS analysis after BrdU incorporation performed on GB8 cells showed that APE treatment did not modify significantly the percentage of cells in S phase, although a faint reduction of cells in S phase was visible after 96 h of APE treatment accompanied by a progressive increase of cells in G2/M phase (Figure 4A,B). Western blot analysis of the cell cycle-associated protein PCNA has demonstrated an increase of expression that was higher in GB8 cells compared to GB7 cells (Figure 5A,B). Interestingly, the test of recovery performance by maintaining the GSC cells in the presence of APE for 120 h and then, after M2 agonist withdrawal, for an additional 72 h, 96 and 120 h in fresh medium without APE, demonstrated that only GB7 cells were able to rescue their proliferation (Figure 5C,D).

**Figure 6:**
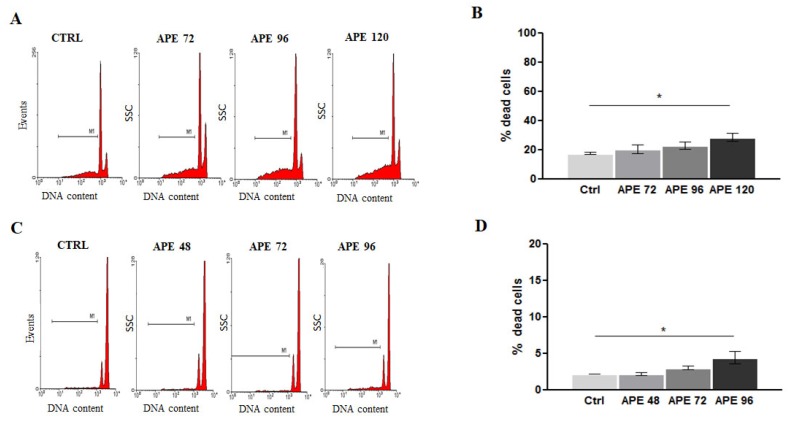
(**A**) Cytometric analysis of dead cells in GB8 cell cultures after 100 μM APE treatment. (**B**) Percentage of apoptotic cells present in GB8 cultures after 100 μM APE treatment. (**C**). Cytometric analysis of apoptosis in GB7 cells after APE treatment. (**D**) Percentage of apoptotic cells present in GB7 cultures after 100 μM APE treatment. (ANOVA test; * *p* < 0.05). The data are the average ±SEM of three independent experiments.

**Figure 7:**
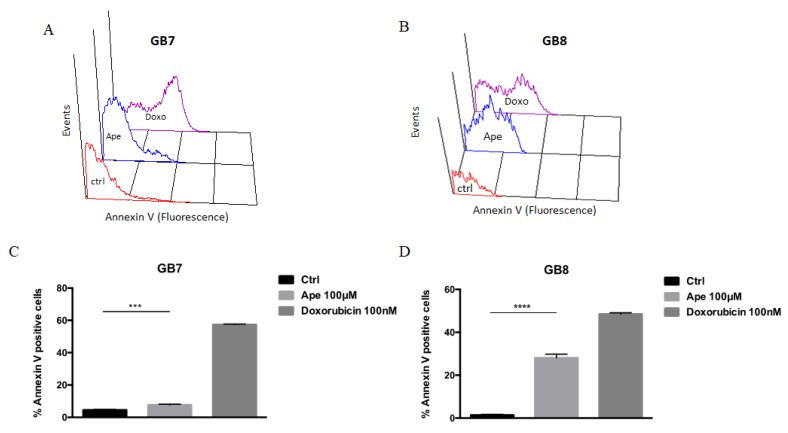
Distribution diagram of Annexin V expression in GB7 (**A**) and GB8 (**B**) cells. Percentage of Annexin V positive cells in GB7 (**C**) and GB8 (**D**) cultures after 100 μM APE treatment. Doxorubicin (100 nM) was used as a positive control. (t-test; APE 72 h vs. Crtl *** *p* < 0.001; **** *p* < 0.0001). The data are the average ±SEM of three independent experiments.

To assess whether the decrease in cell number observed after APE treatment in GSCs was also determined by cell death occurrence, we determined by FACS analysis the fraction of cells with hypodiploid DNA content and higher granularity (SSC) (this fraction was assumed to represent dead cells). This analysis showed that APE treatment is able to increase the percentage of hypodiploid cells in GB8 (Figure 6A,B) and somewhat less in GB7 cultures. On the contrary, the analysis of cell death by FACS analysis after propidium iodide staining in GB7 cells demonstrated a lower percentage of cell death upon APE treatment as compared to GB8 (Figure 6C,D). In order to confirm the apoptotic cell death, the annexin V positive cell number has been evaluated by FACS analysis after 72 h treatment. As shown in Figure 7, APE treatment significantly increased the percentage of annexin V positive cells both in GB7 and GB8 cells. However, the GB8 cells exhibited a higher percentage of annexin V positive cells when compared to GB7 (Figure 7).

### 3.4. Activation of M2 Muscarinic Receptor Differently Induces DNA Damage Effects in GB7 and GB8 Cells

**Figure 8:**
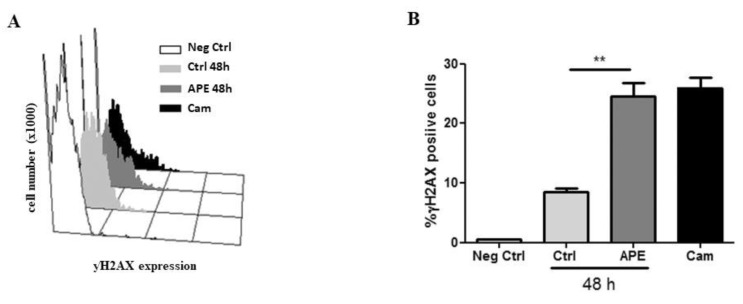
(**A**) Distribution diagram of p-γH2AX expression vs. cell number (×1000). (**B**) Percentage of p-γH2AX positive cells in GB8 cells after 100 μM APE treatment. Camptothecin (5 µM) was used as a positive control. (t-test; APE 48 h vs. Crtl ** *p* < 0.01). The data are the average ±SEM of three independent experiments.

Since a consistent cell death effect was observed in GB8 cells after APE treatment, we investigated its possible causes. Phosphorylated histone γ-H2AX is a marker of cellular response to Double-Strand DNA Breaks (DSB). We used this marker to identify the presence of genotoxic damage induced by APE treatment. Camptothecin treatment was used as a positive control. Flow cytometry analysis on GB7 and GB8 cultures treated with 100 μM APE for 48 h or with 5 μM camptothecin for 24 h, showed that the percentage of the γ-H2AX positive cells increased significantly in GB8 cells after APE treatment, suggesting the occurrence of DNA double-strand breaks induced by M2 agonist treatment (Figure 8A,B). GB7 cells did not exhibit γ-H2AX expression neither in untreated or in APE treated conditions (data not shown).

### 3.5. M2 Modulation Affects Notch1 and EGFR Expression in GB7 Cells

**Figure 9:**
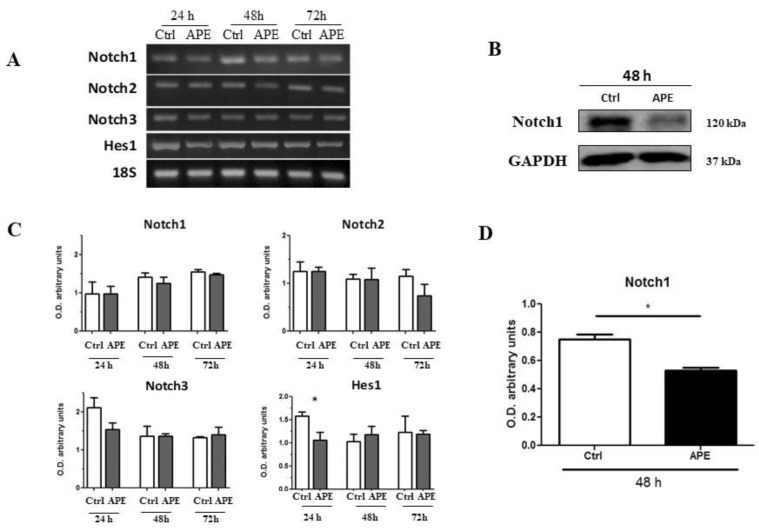
(**A**) RT-PCR analysis for Notch1, Notch2, Notch3, and Hes transcripts in GB7 cells. 18 S was used as a housekeeping gene. (**B**) Notch1 expression by Western blot analysis in GB7 cells. GAPDH was used as the internal reference protein. (**C**) Densitometric analysis of the RT-PCR bands indicated in Figure A normalized with the bands of housekeeping gene 18 S. The data are the average ±SEM of three independent experiments. (**D**) Densitometric analysis of Notch1 immunoreactive bands; Western blot normalized with the bands of the reference protein GAPDH. The data are the average ±SEM of three independent experiments (* *p* < 0.05 t-test).

**Figure 10:**
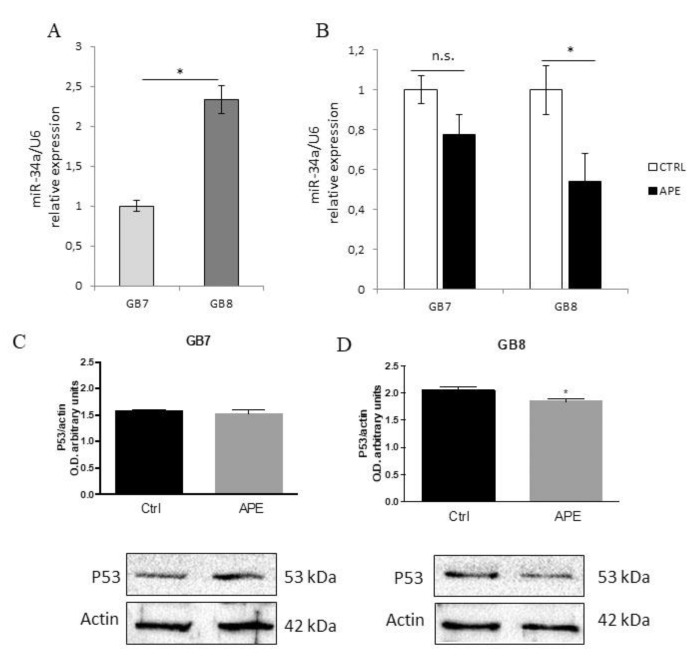
qRT-PCR analysis for miR-34a-5p expression in GB7 and GB8 cells. (**A**) miR-34a-5p levels in untreated GB7 or GB8 cultures. MiRNA relative expression in GB7 was set as one and snRNA U6 used as an internal standard. Data are expressed as the average ±SEM. (N = 3, * *p* ≤ 0.05, t-test). (**B**) miR-34a-5p levels in untreated (CTRL) or APE-treated (100 μM for 24 h) GB7 or GB8 cells. For each cell line, miRNA relative expression in CTRL samples was set as one and snRNA U6 used as an internal standard. Data are expressed as the average ±SEM. (N = 3, * *p* ≤ 0.05, t-test). Western blot analysis of p53 expression in GB7 (**C**) and GB8 (**D**) cells in absence (ctrl) and presence of 100 µM APE (48 h). The graphs above report the densitometric analysis of the p53 immunoreactive bands normalized with the bands of the reference protein β-actin. The data are the average ±SEM of three independent experiments (t-test; * *p* < 0.05).

In order to clarify the mechanisms responsible for the decreased cell proliferation induced by M2 receptor activation observed in particular in GB7 cells, we evaluated Notch-1 transcript and protein expression by RT- PCR and Western blot analyses upon APE treatment (Figure 7). Analysis of Notch genes’ expression by RT-PCR indicated that neither Notch1 or Notch2 and Notch3 were modulated by M2 agonist treatment, although the main Notch target gene, Hes gene, appeared to be negatively modulated by APE at least after 24 h of treatment (Figure 9A). Interestingly, Western blot analysis demonstrated that the expression of Notch1 protein was significantly decreased after 48 h of APE treatment (Figure 9B). Our previous work performed in U87 cells demonstrated that Notch1 protein levels are controlled by miR-34a [25] through p53 activity. In order to explain the decreased Notch1 protein levels after APE treatment, we evaluated the levels of miR-34a in both GSC lines. Interestingly, we observed that both GB7 and GB8 cells express miR-34a (Figure 10), and APE treatment significantly reduced the expression levels in GB8 cells only (Figure 10B). Interestingly, also p53 protein that play a relevant role in miR-34a modulation, albeit expressed in both cell lines, was reduced only in GB8 cells after APE treatment (Figure 10D).

**Figure 11:**
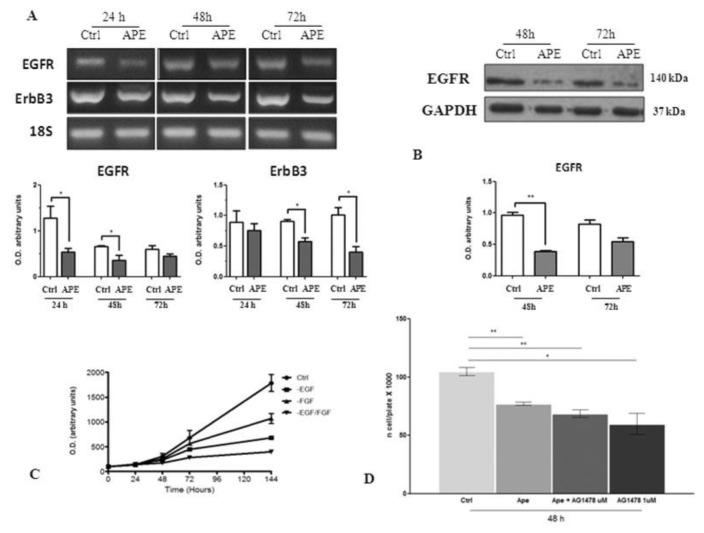
(**A**) RT-PCR analysis of EGFR and ErbB3 receptors in GB7 cells. 18 S was used as a housekeeping gene. The graphs below show the densitometric analysis of the bands of RT-PCR indicated in Fig. A normalized with the bands of housekeeping gene 18 S. The data are the average ±SEM of three independent experiments (* *p* < 0.05 t-test). (**B**) EGFR expression by Western blot analysis on GB7 cells. GAPDH was used as an internal reference protein. The graph below shows the densitometric analysis of the bands of Western blot analysis for EGFR normalized with the bands of the reference protein GAPDH. The data are the average ±SEM of three independent experiments (** *p* < 0.01 t-test). (**C**) MTT assay performed on GB7 cells maintained in complete culture medium containing EGF and FGF (ctrl) either in the absence of FGF, or of EGF, or in absence of both growth factors. (**D**) MTT assay performed after 48 h of treatment with 100 μM APE or 1 μM of TKI AG1478 inhibitor (t-test; * *p* < 0.05; ** *p* < 0.01). The data are the average ±SEM of three independent experiments performed in triplicate.

Another pathway involved in GBM growth and survival is EGFR signaling [24]. In order to understand whether this pathway was also affected by M2 receptor activation, we evaluated EGFR and ErbB3 transcripts and protein expression levels in GB7 cells by RT-PCR and Western blot analysis, respectively. RT-PCR analysis showed that the EGFR and ErbB3 receptor expressions were negatively modulated following APE treatment (Figure 11A). This result was also confirmed by Western blot analysis that showed that M2 receptor activation negatively modulated the EGFR receptor expression (Figure 11B).

To confirm the involvement of EGFR in cell proliferation, we cultured GB7 cells in the presence or absence of EGF. MTT assay resulted in a significant decrease of cell number when GB7 cells were maintained without EGF (Figure 11C). The treatment with (EGFR) tyrosine kinase inhibitor (TKI) tyrphostin AG1478 moreover demonstrated that the GB7 cell proliferation is mainly dependent on EGFR receptor activity. Indeed, the inhibition of EGFR pathway by AG1478 induced a significant reduction of cell growth comparable to APE treatment. Additionally, co-treatment of GB7 cells with APE and AG1478 did not show any synergic effect of the two drugs (Figure 11D).

## 4. Discussion

Muscarinic receptors are classical G-protein coupled receptors involved in many physiological functions. In the last years, several evidences have described an involvement of these receptors in different pathological conditions, both in neuronal and non-neuronal tissues [26,27]. Moreover, the contribution of muscarinic receptors in tumor progression has also been demonstrated and the use of agonists or antagonists for different cholinergic receptor subtypes is emerging as therapy for different pathologies [11,26,27,28]. Previously it has been demonstrated the ability of M2 receptor to counteract tumor cell growth and survival both in GBM and in GSCs [16,17,18,19], in neuroblastoma [29] and in urothelial bladder cells [30].

Here we have extended these studies by evaluating the mechanisms downstream of the M2 receptor activation by orthosteric agonist APE in the modulation of cell cycle progression and reduced survival in GSCs. For this purpose, we have selected two GSC lines derived from human biopsies (GB7 and GB8 cells) that expressed different levels of M2 muscarinic receptor, in order to further compare the effects mediated by APE on GSCs expressing different amounts of M2 receptor. Previous analysis of cell viability demonstrated that APE triggered a decrease of the cell growth in a time- and dose-dependent manner in GB7 cells. In GB8 cells, instead, the decrease on the cell number is evident only after treatment with a high dose (100 μM) of APE [18]. In order to clarify whether the APE-induced decrease in cell number was dependent on impaired cell proliferation, the cell cycle progression was evaluated by FACS analysis and BrdU incorporation. The results indicated a different behavior of the two GSC lines upon APE treatment. In fact, GB7 cells exhibited a decrease of cells in S phase with their accumulation in G2/M and a non-substantial change in G1 phase, confirming an APE-induced decrease in cell proliferation. Conversely, in GB8 cells, M2 stimulation produced a significant change in percentage of cells in S phase only at longer exposure (96–120 h) of APE treatment, and a cell accumulation in G2/M with a progressive reduction of cells in G1 phase was evident. To better evaluate the proliferative block in the two cell lines, PCNA expression was assessed by Western blot analysis. Unexpectedly, PCNA expression increased after APE treatment in both cell lines. This could be explained as a consequence of the accumulation of cells in G1-G2/M phases rather than an increased proliferation. However, to understand if the proliferative arrest was reversible, we performed a rescue analysis of the cell proliferation after 120 h of treatment with APE. It is evident that GB7 cells are able to recover cell proliferation after removing APE from the culture medium, while GB8 cells are unable to resume proliferation. It is evident that the two cell lines behave differently, and in GB7 cells the activation of the M2 receptor causes reversible arrest while in GB8, proliferative arrest is permanent at least at the doses used. Interestingly, FACS analysis and propidium iodide staining demonstrated that the APE treatment caused an increase of the number of hypodiploid cells overall in GB8 cells. Conversely, in GB7 cells, APE induced a lower percentage of cell death compared with GB8 cells. The apoptotic cell death is also confirmed by the analysis of annexin V. Moreover, similarly, as observed by propidium iodide analysis, GB8 cells present a higher percentage of annexin V positive cells than GB7 cells. These results suggest that the decrease in cell number observed following APE treatment was probably due to reduced cell proliferation in GB7 cells while in GB8 cells it may be dependent on decreased cell survival. The cell death effects observed in GB8 cultures were caused by DNA damage, in fact, we found a significant increase of the number of γH2AX-positive cell lines. The apoptotic effects observed in GB8 cells and the presence of APE-induced genotoxic effects in these cells may be explained by the occurrence, only in GB8 cells, of a mutation in p53 at the level of Arg342 [18]. The presence of the mutated form of p53 protein may therefore contribute to confer higher susceptibility of GB8 cells to an APE toxic effect. In fact, the cells presenting the genotoxic damage may continue in the mitotic division, and in the presence of mutated p53, failing to resolve the DNA damage, they go into cell death. On the other hand, GB7 cells presenting p53 wild type may be able to resolve possible APE-induced genotoxic effects. However, these results are in agreement with that previously reported for GBM cell lines U87 and U251. Indeed, similar to that observed in GSCs, U251 cells that presented p53 mutated showed APE-induced cytotoxic and genotoxic damage and increased cell death compared to U87 cells that presented wild type p53 genotype [31].

In order to explain the significant decrease of cell proliferation occurring in GB7 cells, we also investigated the ability of APE to impair Notch/EGFR pathways. In fact, our previous studies performed in GBM cell lines have indicated the ability of M2 receptors to negatively regulate Notch and EGFR pathways [25]. Similarly, in the present study we described that APE treatment caused a significant decrease of Notch1 protein levels. This decrease appeared to be caused by post-transcriptional control produced by M2 receptor stimulation on Notch expression, considering that the mRNA levels of Notch did not appear significantly modified by APE treatment. However, the analysis of miR-34a-5p, one of the main miRNA involved in the post-transcriptional regulation of Notch1 [31], revealed its expression in both cell lines, and the activation of the M2 receptor seems to downregulate miR-34a expression preferentially in GB8 than in GB7 cells, indicating that miR-34a-5p is not involved in the post-transcriptional control of Notch1 in these GSC cell line. The expression of miR-34a is regulated by p53 [25]. For this reason, we have also analyzed the p53 expression in both GSC cell lines, observing that p53 is expressed both in GB7 and GB8 cells and that the APE treatment caused a significant decrease of p53 expression but only in GB8 cells. Therefore, the downregulation of miR-34a after APE treatment may be dependent on the reduced expression of p53, at least in GB8 cells.

Notch1 positively regulates EGFR transcription [10,32]; therefore, the downregulation of EGFR observed in GB7 cells could also be explained through APE-induced Notch-1 downregulation. The role played by Notch and EGFR in GSCs proliferation might be relevant. In fact, the absence of EGF in the culture media or the inhibition of EGFR activity by TKI AG1478 inhibitor, indicated that the absence of EGF or the block of the pathway downstream EGFR, significantly impaired GB7 cell proliferation.

## 5. Conclusions

The reported data confirm that the M2 agonist APE is able to counteract cell proliferation and survival in GSCs. The results indicate that the M2 receptor activation may differently act in GSCs with wild type and mutant p53 genotypes. In GB7 cells bearing the wild type p53 genotype, the M2 receptor activation may activate a molecular circuitry based on the cross-talk between M2 receptor and the Notch-1/EGFR pathways [25].

This circuitry is deregulated in the GB8 cells characterized by p53 mutated and negatively modulated by APE. In these cells, M2 receptor activation may cause a genotoxic effect probably mediated by ROS production that impair GB8 cell survival [31].

In both cases, M2 receptor appears as an interesting therapeutic tool for glioblastoma therapy. Although the level of expression of M2 receptors in GSC obtained by different patients is heterogeneous (see Figure 1) and the therapeutic potential of the M2 receptor may be conditioned by the genetic background (i.e., p53 wild type or mutated) of each patient, the last advances in the treatment of these tumors showed the development of personalized therapies to be the most efficient way to pursue this. In this panorama, our results represent a remarkable step in the perspective of a new possible therapeutic approach for GBM treatment. 

## Supplementary Materials

The following are available online at https://www.mdpi.com/2073-4409/9/3/657/s1, Figure S1: Western blot analysis for M2 receptor expression in GB7 cells maintained in adherent and neurophere conditions. Rat brain was used as positive control. Beta- actin was used as reference protein. Figure S2: Espression of stemness markers in GB8 cell line. (**A**) Analysis by RT-PCR of Nestin and Sox2 transcripts in GB8 cell line in adherent and sphere conditions. U87 stable cell line was used as negative control. 18s was used as housekeeping gene. (**B**) Immunocytochemistry analysis of Nestin (green) and Sox2 (red) protein expression in GB8 cells. DAPI was used to counterstain the nuclei (×100).
