# Supplementary material for: Cross Interaction between M2 Muscarinic Receptor and Notch1/EGFR Pathway in Human Glioblastoma Cancer Stem Cells: Effects on Cell Cycle Progression and Survival
Source: Cells. 2020 Mar 9;9(3):657. doi: 10.3390/cells9030657 (PMC7140674; doi:10.3390/cells9030657)
Supplement: Supplementary file 1 [file cells-09-00657-s001.zip › cells-696856-supplementary.pdf]

cells

MDPI

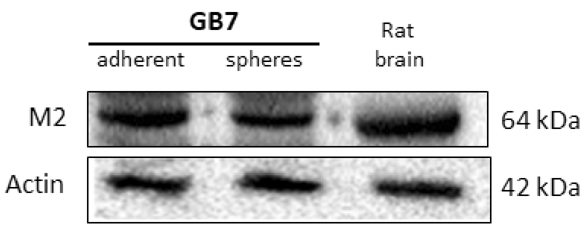

Supplementary Figure 1. Western blot analysis for M2 receptor expression in GB7 cells maintained in adherent and neurophere conditions. Rat brain was used as positive control. Beta- actin was used as reference protein.

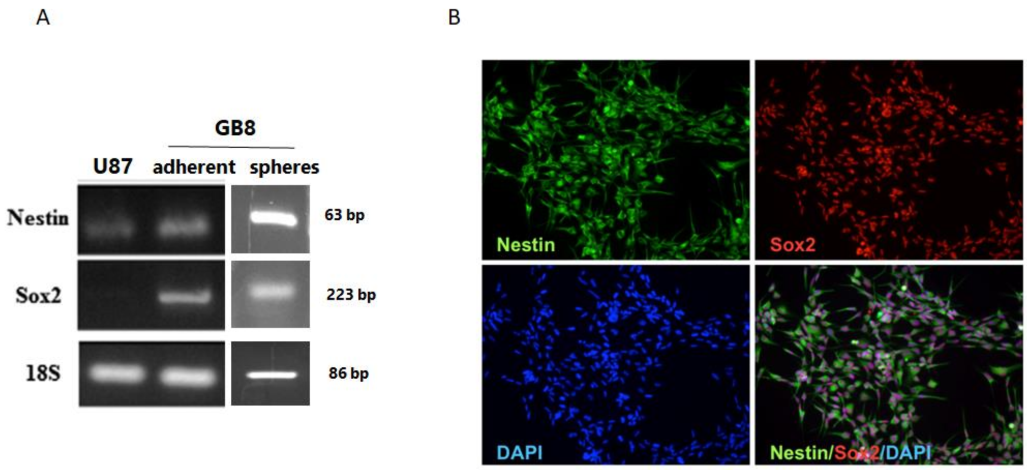

Supplementary Figure 2. Espression of stemness markers in GB8 cell line. (**A**) Analysis by RT-PCR of Nestin and Sox2 transcripts in GB8 cell line in adherent and sphere conditions. U87 stable cell line was used as negative control. 18 s was used as housekeeping gene. (**B**) Immunocyochemistry analysis of Nestin (green) and Sox2 (red) protein expression in GB8 cells. DAPI was used to counterstain the nuclei (100x)
